# An Unexpected Occurrence of Malignancy in a Patient After a Fontan Operation

**DOI:** 10.7759/cureus.26160

**Published:** 2022-06-21

**Authors:** Julian Yet Kwong Horman, Michael Schultz

**Affiliations:** 1 Internal Medicine - Pediatrics, Penn State Health Milton S. Hershey Medical Center, Hershey, USA

**Keywords:** metastatic germ cell tumor, nonseminomatous extragonadal germ cell tumor, dextro-transposition of great arteries, hematology-oncology, adult congenital heart disease (achd)

## Abstract

An 18-year-old male with complex single ventricle physiology status post Fontan and Kawashima procedures who presented with progressive dyspnea was found to have severe acute respiratory syndrome coronavirus 2 (SARS-CoV-2), rhinovirus, and a new retroperitoneal mass. Biopsy of the retroperitoneal mass revealed a mixed germ cell tumor with areas consistent with choriocarcinoma. Imaging showed metastatic disease, including to the lungs which ultimately led to worsening respiratory failure that required intubation and ultimately, death.

## Introduction

Congenital heart disease (CHD) is a broad category of diseases that carries significant mortality and morbidity. With recent advances in medical and surgical management, CHD patients overall have benefited from an increased life expectancy. The exact heart lesion is vital in the overall prognosis. Ventricular septal defects are the most common congenital heart lesion, occurring in 42 out of 10,000 births in the United States [1]. The 97% of patients with non-critical CHD are expected to live to one year of age and 95% are expected to survive to 18 years of age. Critical CHDs have lower survival rates, with 75% expected to survive to one year of age and 69% expected to survive to 18 years of age. Survival of critical CHD patients is improving though, between 1979 and 1993 the expected survival to one year was 67%. With advances in medical care, survival to one year increased to 83% between 1994 and 2005 [2]. 

With more CHD patients living into adulthood, their complications have continued to evolve. The CHD patients are certainly at risk for complications secondary to their cardiac lesion but are also at risk for complications related to normal aging. Unfortunately, in this patient population, common complications that can occur with aging may be exacerbated by their underlying cardiac anomaly. Adult congenital heart disease (ACHD) patients are already at higher risk for arrhythmias, ventricular dysfunction, and heart failure so the development of acquired heart disease with aging can be detrimental [3].

The CHD can lead to the dysfunction of multiple other organ systems. Liver, kidney, and lungs are often impacted by CHD, around 50% of ACHD patients exhibit abnormal glomerular filtration rates and 40% have abnormal lung function testing. Heart failure is the most common cause of death, with pneumonia, sudden cardiac death, and malignancy as other leading causes of mortality [4].

## Case presentation

An 18-year-old male with abdominal heterotaxy, transposition of the great vessels, and atrioventricular (AV) canal defect repaired with a Kawashima and Fontan procedure presented to an outside facility due to progressive shortness of breath. About two weeks prior to presentation he developed a dry, non-productive cough as well as progressive exertional dyspnea. Due to the cough and worsening dyspnea he presented for evaluation.

**Figure 1:**
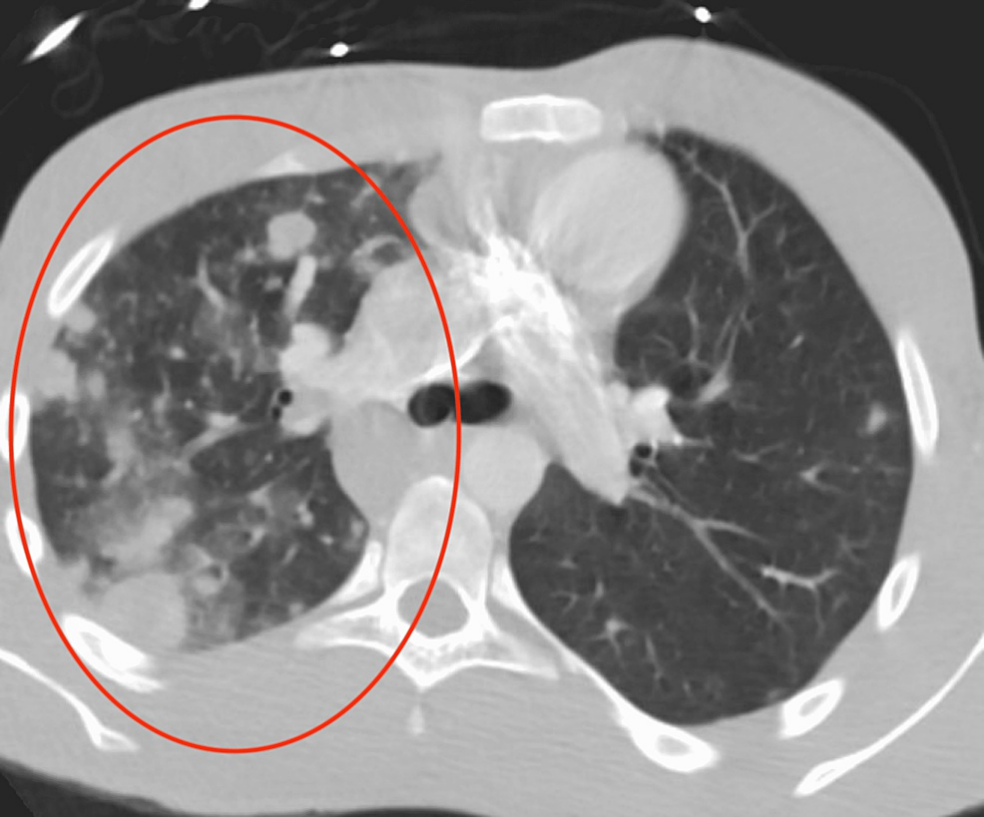
Initial thoracic CT. Red circle showing numerous nodules in the lung

At the outside hospital, he was hypoxic with oxygen saturations in the 80 %s. At baseline, the patient is very active with saturations normally greater than 92%. He was started on supplemental oxygen requiring up to 6 L to maintain saturations greater than 90%. He was tested for severe acute respiratory syndrome coronavirus 2 (SARS-CoV-2) via polymerase chain reaction (PCR) testing which was negative. A thoracic CT scan was performed due to his dyspnea and hypoxia which showed multiple lung nodules (Figure 1).

**Figure 2:**
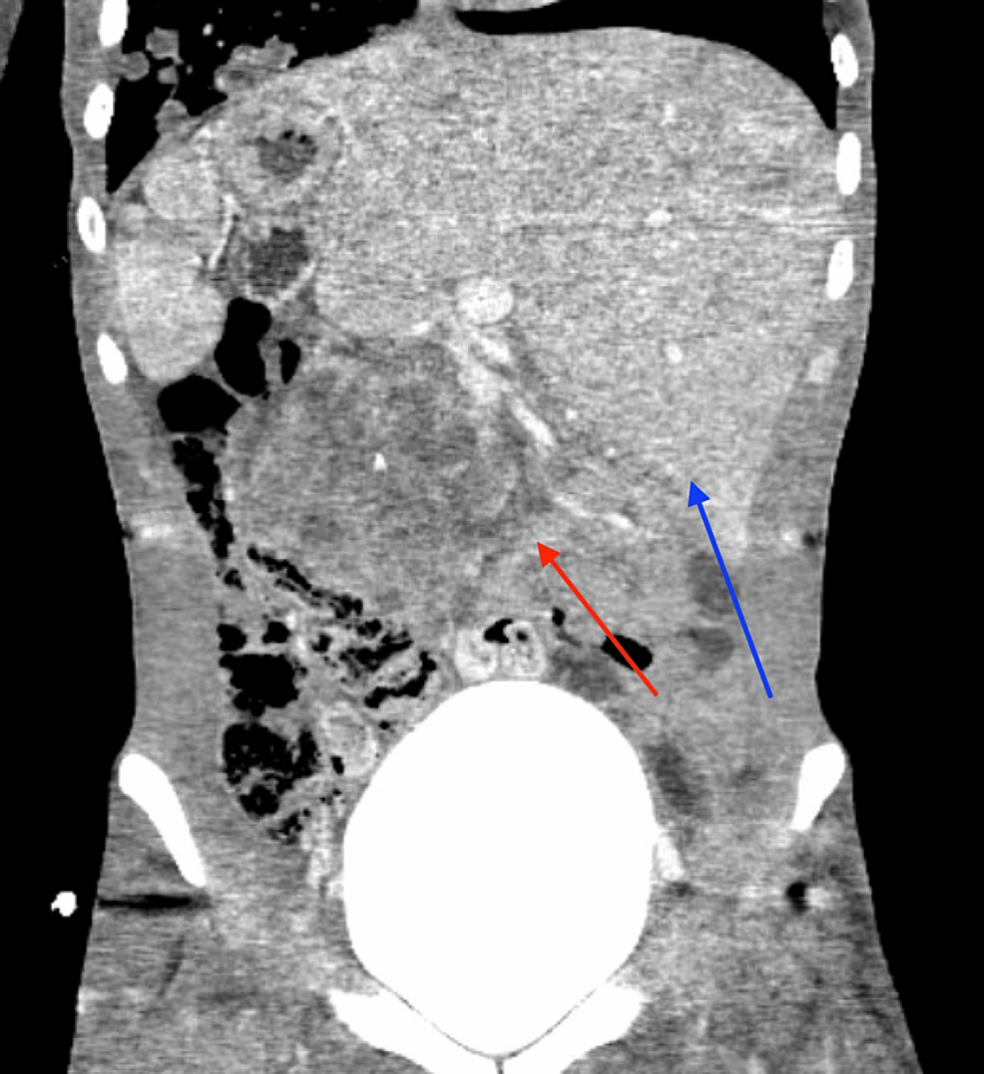
Abdominal CT. Red arrow pointing at the 9-cm retroperitoneal mass Blue arrow pointing to the liver on the left side of the abdomen consistent with the patient's abdominal heterotaxy

He was also incidentally found to have right-sided hydronephrosis on the chest CT, so a dedicated CT of the abdomen was performed. The CT of the abdomen revealed a large, 9-cm retroperitoneal mass compressing the right ureter with subsequent right hydronephrosis (Figure 2). 

There was also an enhancing lesion within the dome of the liver measuring 2.3 cm. Despite the findings on the abdominal CT, his physical exam did not reveal any abnormal abdominal findings. Due to the imaging findings, he was transferred to a large quaternary care center.

Upon arrival at the quaternary care center, he tested positive for SARS-CoV-2 and rhinovirus via respiratory viral panel PCR. He was started on remdesivir to treat the SARS-CoV-2 infection to complete a five-day course. His complete blood count revealed a hemoglobin of 8.4 g/dL with a normal white blood cell count and platelet count. His iron panel was consistent with iron deficiency anemia but he did not have any signs of bleeding on physical exam or per his history. His liver function tests were all within normal limits as well. Interventional radiology placed a right nephrostomy tube to help alleviate the ureteral obstruction. Urology was consulted and recommended obtaining lactate dehydrogenase (LDH), alpha-fetoprotein (AFP), and beta-human chorionic gonadotropin (b-HCG) levels to assess for malignancy due to the retroperitoneal mass. The LDH returned elevated at 420 U/L (normal between 135 and 250 U/L), AFP was normal at 1.0 ng/mL (normal <8.4 ng/mL), and the b-HCG was significantly elevated at 60,338 milli-international units (mIU)/mL (normal <5 mIU/mL) which raised concern for a germ cell tumor.

**Figure 3:**
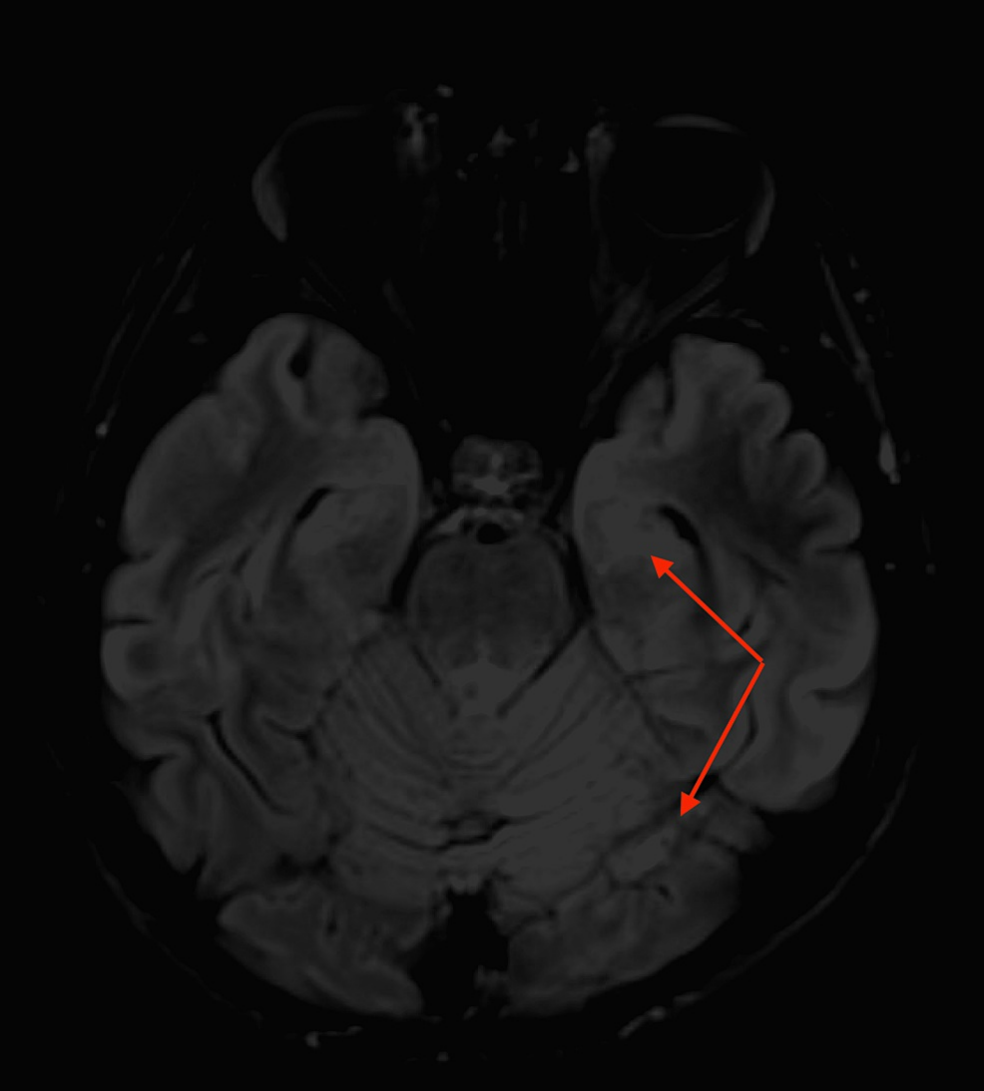
MRI of the brain. Red arrows pointing to lesions concerning possible acute embolic infarctions versus brain metastases

Oncology was then consulted due to the concern for a germ cell tumor. They recommended obtaining an ultrasound of the testes and an MRI of the brain for staging purposes. The testicular ultrasound did not show any evidence of testicular masses. The MRI of the brain revealed numerous areas with restricted diffusion in the lateral frontal, parietal, and left temporal lobes as well as a few scatter foci of restricted diffusion in the bilateral occipital lobes which raised concern for acute embolic infarcts or metastatic disease (Figure 3).

He was started on a therapeutic heparin drip due to the possible embolic infarcts. A hypercoagulability workup was performed, including protein C, protein S, antithrombin III, beta-2-glycoprotein antibody, anticardiolipin antibody, lupus anticoagulant, and homocysteine all of which returned normal. He was then transitioned to warfarin for ongoing anticoagulation. 

Cardiology was consulted and followed the patient through his hospital stay. As the etiology of his hypoxia was likely related to the numerous lung lesions, a cardiac evaluation for his hypoxia was not felt to be necessary. A transthoracic echocardiogram (TTE) was considered due to the possible embolic infarctions of the brain but was ultimately deferred. Due to his positive SARS-CoV-2 status, the available ultrasound machines were unable to obtain high-quality images, therefore, it was decided to delay obtaining a TTE until he was out of SARS-CoV-2 isolation and higher-quality ultrasound machine could be utilized. 

His supplemental oxygen requirements continued to fluctuate, requiring between 1 L and 5 L of supplemental oxygen. Once his respiratory status stabilized, the retroperitoneal mass was biopsied which was consistent with a mixed germ cell tumor with components of choriocarcinoma. A few days after the biopsy was performed his respiratory status worsened, requiring a high flow nasal cannula to maintain adequate oxygen saturations. His continued oxygen requirement was felt to be multifactorial in the setting of the likely metastatic lung nodules and fluid overload. The SARS-CoV-2 was not felt to be a large contributor to his persistent hypoxia as he completed a full course of remdesivir and the SARS-CoV-2 PCR was negative which raised suspicion that the positive respiratory viral panel PCR was indicative of a past infection. He was started on IV furosemide 20 mg twice daily which resulted in brisk diuresis and his oxygen requirement improved, allowing him to transition from high flow nasal cannula to nasal cannula. He continued to require supplemental oxygen via nasal cannula, therefore, the decision was made to start treatment for the mixed germ cell tumor.

**Figure 4:**
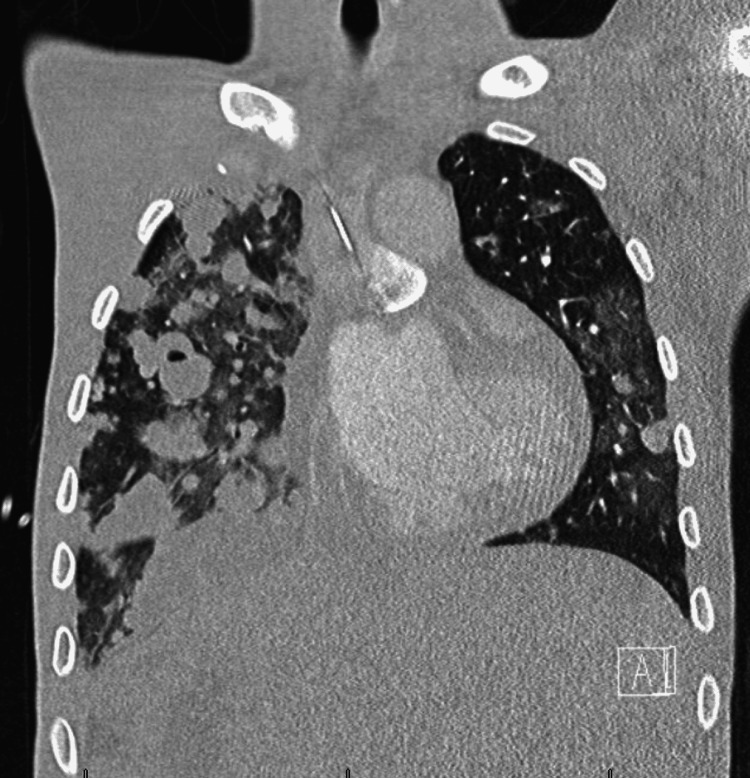
Repeat thoracic CT. CT chest showing innumerable lung lesions, which is worse than the previous studies raising concern for progressive metastatic disease

He was started on chemotherapy with etoposide, ifosfamide, and cisplatin. He soon developed hemoptysis and his respiratory status worsened, again requiring a high flow nasal cannula. A CT of the chest did not show any evidence of pulmonary hemorrhage but did show worsening metastatic pulmonary disease, nonetheless, the warfarin was discontinued (Figure 4).

Unfortunately, his respiratory status continued to worsen despite high flow nasal cannula so he was trialed on bilevel positive airway pressure (BiPAP) but he did not tolerate it well, so he was transitioned back to high flow nasal cannula for a short period of time but then was ultimately intubated. Soon after intubation, he developed hypotension that was likely due to the increased intrathoracic pressure from intubation. His blood pressure did not respond to aggressive fluid administration and required norepinephrine, vasopressin, and epinephrine to maintain adequate blood pressure.

A family meeting was held due to his rapidly worsening clinical condition and the decision was made to transition the patient to comfort care. He ultimately passed away later that day. 

## Discussion

Choriocarcinoma is a rare malignancy that most commonly occurs in females but males can also be affected. Choriocarcinoma is classified as gestational and non-gestational, with males affected by non-gestational choriocarcinoma although usually as a mixed germ cell tumor with choriocarcinoma being a component. Choriocarcinoma tends to metastasize, often to the lungs, liver, gastrointestinal tract, and brain. Patients often present to medical care due to symptoms related to metastatic diseases, such as hemoptysis. Gestational choriocarcinoma generally has a good prognosis, with low-risk gestational choriocarcinoma carrying almost 100% survival with treatment and high-risk choriocarcinoma having a survival rate of 91%-93% with treatment. In males with mixed germ cell tumors, increasing amounts of choriocarcinoma components lead to a worse prognosis, b-HCG that is greater than 50,000 mIU/mL also has a worse prognosis [5].

The CHD predisposes to numerous systemic complications. Malignancy is an increasingly common cause of morbidity and mortality in this patient population. Some studies have suggested that CHD patients have a significantly elevated malignancy risk as compared to the general population. A Swedish showed that CHD patients have an incidence of malignancy that was twice that of healthy matched controls. Furthermore, patients with complex heart lesions, such as conotruncal defects, had an even higher risk of malignancy with a hazard ratio of 2.29 [6].

The exact reason for the increased risk of malignancy in CHD patients is unclear, although it is likely multifactorial. It has been postulated that many of the same genetic and environmental risk factors that may lead to CHD may also increase the risk for malignancy. One study evaluated the correlation between children and young adults with CHD and lymphoma. They concluded that there may be a positive correlation with CHD and lymphoma and that the increased lymphoma risk in CHD patients could suggest a shared developmental origin [7]. The increased incidence of CHD and malignancy seen with trisomy 21 patients may also offer further evidence of an environmental and genetic link.

Another risk factor for malignancy in CHD patients is ionizing radiation exposure. One of the main considerations of many common imaging modalities is radiation exposure and subsequent malignancy risk. The CHD patients often undergo multiple procedures that increase their radiation exposure. One study evaluated the malignancy rates of CHD patients who underwent multiple procedures requiring ionizing radiation compared to the malignancy rates of CHD patients who underwent one or fewer procedures requiring ionizing radiation. They found that ionizing radiation was associated with cancer in CHD patients, with a higher risk of malignancy seen in patients with higher exposure to ionizing radiation [8]. While it is not clear the exact amount of ionizing radiation the patient in this case received, he likely received a significant amount due to his complex cardiac lesions requiring numerous interventions. 

While there seems to be an increased risk of malignancy in CHD patients it is not clear if current malignancy screening guidelines are sufficient for the ACHD population. Despite the increased malignancy risk, ACHD patients may undergo routine cancer screening at lower rates than their non-CHD counterparts. One study evaluated cancer screening rates in ACHD patients and found that female ACHD patients had significantly lower rates of Pap smears and mammography than non-ACHD women [9]. While it remains to be seen if current cancer screening guidelines are sufficient for CHD patients, it is important that CHD patients continue to undergo regular cancer screening. 

## Conclusions

The CHD encompasses numerous cardiac pathologies, each having its own set of complications. As medical and surgical care has evolved and improved, so has the life expectancy of CHD patients which has led to new discoveries regarding long-term complications of CHD. While cardiac complications continue to be the leading cause of morbidity and mortality, malignancy has also become common. While CHD patients seem to have an increased risk of malignancy, the exact mechanism is not understood but it is likely multifactorial.
